# Degradation-Aware Deep Learning Framework for Sparse-View CT Reconstruction

**DOI:** 10.3390/tomography7040077

**Published:** 2021-12-09

**Authors:** Chang Sun, Yitong Liu, Hongwen Yang

**Affiliations:** Science of Information and Communication Engineering, Beijing University of Posts and Telecommunications, Beijing 100876, China; sunchang1996@bupt.edu.cn (C.S.); yanghong@bupt.edu.cn (H.Y.)

**Keywords:** sparse-view CT reconstruction, degradation-aware, deep learning, frequency domain, image domain

## Abstract

Sparse-view CT reconstruction is a fundamental task in computed tomography to overcome undesired artifacts and recover the details of textual structure in degraded CT images. Recently, many deep learning-based networks have achieved desirable performances compared to iterative reconstruction algorithms. However, the performance of these methods may severely deteriorate when the degradation strength of the test image is not consistent with that of the training dataset. In addition, these methods do not pay enough attention to the characteristics of different degradation levels, so solely extending the training dataset with multiple degraded images is also not effective. Although training plentiful models in terms of each degradation level can mitigate this problem, extensive parameter storage is involved. Accordingly, in this paper, we focused on sparse-view CT reconstruction for multiple degradation levels. We propose a single degradation-aware deep learning framework to predict clear CT images by understanding the disparity of degradation in both the frequency domain and image domain. The dual-domain procedure can perform particular operations at different degradation levels in frequency component recovery and spatial details reconstruction. The peak signal-to-noise ratio (PSNR), structural similarity (SSIM) and visual results demonstrate that our method outperformed the classical deep learning-based reconstruction methods in terms of effectiveness and scalability.

## 1. Introduction

In the past fifty years, computed tomography (CT) has been broadly applied in clinical diagnostics, nondestructive testing and biological research due to the high resolution and high sensitivity of CT images [1]. However, its high radiation dose can lead to headaches or even cancer and leukemia in severe cases [2]. In addition, a long scanning time and high scanning frequency can further increase hazards [2]. One straightforward way to tackle this problem is by reducing the number of X-ray photons emitted by the detector by decreasing the currents on the X-ray tube, but this may result in heavy noise interference and undesired artifacts in CT images. Another alternative solution is to lower the frequency of X-ray scanning and accelerate the acquisition. Sparse-view CT and limited-angle CT reduce the frequency of measurement by sparsely projecting the object and controlling the projection angle to a limited extent, respectively. Nevertheless, severe streak artifacts and directional artifacts in reconstructed CT images inevitably occur due to insufficient data collection. Therefore, the study of low-dose CT reconstruction has received extensive attention from researchers.

CT reconstruction methods can be broadly classified into three categories, i.e., sinogram-domain reconstruction, iterative reconstruction (IR) and image-domain reconstruction. Sinogram-domain methods perform denoising, the removal of artifacts and interpolation in sinogram data by utilizing traditional filtering algorithms [3,4,5], dictionary-based approaches [6] and deep learning-based methods [7]. Filtering algorithms have the advantages of their computation cost and reconstruction speed but fail to achieve satisfying performance when the raw data are severely lacking. On the other hand, dictionary-based and deep learning-based approaches suffer from undesired artifacts or over-smoothing in CT images due to indirect processing in the sinogram-domain. In contrast, by iterative correction and reconstruction, IR methods such as the algebraic reconstruction technique (ART) [8], simultaneous algebraic reconstruction technique (SART) [9] and simultaneous iterative reconstruction technique (SIRT) [10] can produce CT images with better quality and less noise and artifacts. Moreover, under the guidance of the compressed sensing (CS) theory [11,12], the prior knowledge has been employed to constrain the solution space, such as those of ART total variation (ART-TV) [13], edge persevering TV (EPTV) [14], adaptive-weighted (AwTV) [15], non-local means (NLM) [16,17,18] and low-rank [19]. Aided by the prior knowledge, IR methods achieve favorable performance while sacrificing large computation resources. Another CT reconstruction approach is applying an image processing method in the image domain which is similar to natural image processing [20,21,22]. The main convenience of the image domain is that it does not require raw sinogram data.

Recently, deep learning methods have been particularly influential in image-domain CT reconstruction. Many CNN-based algorithms have outperformed IR methods by a large margin at a specific degradation level [23,24,25]. Unfortunately, as a result of supervised learning tailored for a single degradation strength, these fail to obtain a favorable reconstruction performance on other degradation levels due to the identical processing of all corrupted data. An viable way to address this problem is training plentiful models to target each degradation level, however, this may be greatly challenging to deploy in practice due to the large consumption of training computation and the growth of parameter storage. Furthermore, with the extension of degradation levels, the cost of training and parameters linearly increase, which is not scalable and practical in real applications. On the other hand, some researchers have proposed mitigating this problem by mixing multiple data at possible degradation levels to construct a training dataset. Chen et al. proposed a RED-CNN+ model which was trained on a mixed dataset including three different blank scan photons [25]. Han et al. took advantage of the wavelet transform and investigated a tight-frame U-Net [26] structure which was trained on filtered back projection (FBP) restored images from 60, 120 and 240 sparse views [27]. Xie et al. presented Improved GoogLeNet to remove streak artifacts, which was also trained on FBP restored images from 60 and 120 views [28]. The experiments showed that they can achieve better robustness than training with a single degradation level. Nonetheless, these methods do not pay sufficient attention to the difference in degradation information and perform the implicit learning of degradation attributes. Therefore, these methods do not have sufficient capacity to handle multiple degradations. The reconstructed CT images usually have less textural and structural details in the low degradation level (such as 240 sparse views) and more unexpected artifacts in the high degradation level (such as 60 sparse views). In this study, we focused on investigating a degradation-aware deep learning framework to enhance the robustness of low-dose CT reconstruction at multiple degradation levels.

**Figure 1:**
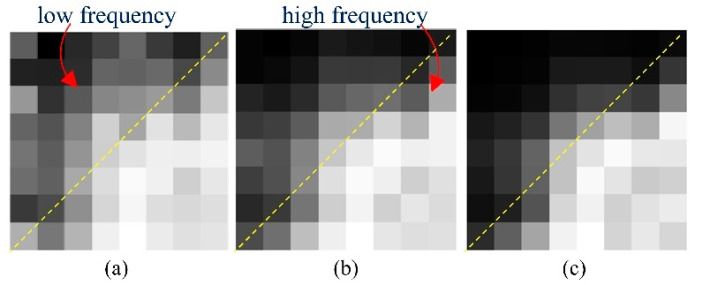
Average reconstruction error of 64 DCT frequencies with different degradation levels. Each pixel represents the MSE between FBP reconstruction and the ideal CT image at the corresponding frequency. The 300 evaluated images were selected from The Cancer Imaging Archive [29,30] for different body parts: (**a**) reconstruction error of 60 views; (**b**) reconstruction error of 120 views; and (**c**) reconstruction error of 240 views.

The key to tackling this problem is to seize the distinction of sparse-view CT degradations and instruct the model to understand it explicitly. According to our analysis, the characteristic of degradation is not only displayed in the image domain, but is also distinctly presented in the frequency domain. Figure 1 shows the reconstruction error in 64 DCT frequency components between FBP reconstruction results and ideal CT images. Low frequencies are on the top–left and high frequencies are on the bottom–right. It is clear from the figure that for the low degradation level (240 views), the difference between the reconstructed images and ideal images is mainly in the high frequencies, which we should mainly focus on in the restoration process. In contrast, in high degradation levels (60 views), the error spans in both low and high frequencies, illustrating the significance of general reconstruction on all frequencies. This result motivated us to attempt to understand the disparity of degradation in both the frequency domain and image domain. To summarize, the main contributions of this study are as follows:

- A novel degradation-aware deep learning framework for sparse-view CT reconstruction is proposed. The proposed framework overcomes the disadvantage of weak generalization at multiple degradation levels of previous single-degradation methods. In addition, it is beneficial for extending to more degradation levels without the growth of training parameters. Experimental results have shown the effectiveness and robustness of the proposed framework.
- A frequency-domain reconstruction module is proposed. It conducts a frequency-attention mechanism to adaptively analyze the disparity of degradation levels by employ distinct operations to each frequency. The experiments described herein illustrate its satisfactory performance on artifact removal and intensity recovery.
- An image-domain module is proposed to further capture the image space degradation characterization from the frequency-domain reconstruction results. This produces a critical-map to emphasize the contour pixels with high reconstruction errors. The experiments show the favorable achievement of the aid of an image-domain module in the structure preservation and edge enhancement.

## 2. Materials and Methods

### 2.1. Network Structure

**Figure 2:**
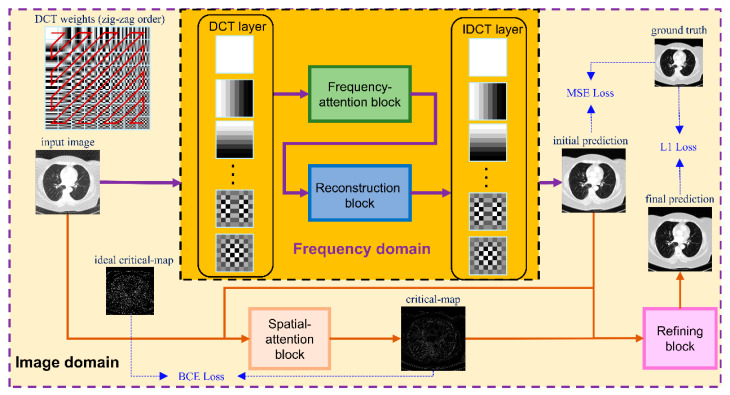
Proposed framework. The whole network contains two modules—one in the frequency domain and one in the image domain. The input image is first deposed into 64 frequency components by a DCT layer and then passes through a frequency-attention block and a reconstruction block. After that, the initial prediction of the ideal CT image is produced by an IDCT layer and is sent to a spatial-attention block in the image domain together with the input image. The output of the spatial-attention block is a critical-map, which is used for the guidance of refining the block to finally predict the ideal image.

The overall framework is shown in Figure 2. The network consists of two modules—one in in frequency domain and one in spatially domain. The frequency-domain module performs a reconstruction procedure in the frequency domain and predicts an initial reconstruction result while the objective of the image-domain module is to conduct fine restoration based on the initial reconstruction of the frequency-domain module. The details of these modules were illustrated in the following sections.

### 2.2. Frequency-Domain Module

A frequency domain reconstruction module was investigated to recover the frequency components which are composed of a DCT layer, a frequency-attention block, a reconstruction block and an IDCT layer. In practice, the input image is first deposed into *N*^2^ DCT frequencies. Given an input CT image *f*(*x*_1_,*x*_2_) of size *H* × *H*, we first split it into blocks without overlapping, and we then we conducted the DCT transform on each block $f_{b}\left(x_{1},x_{2}\right),b=1,2,\ldots ,{\left(\frac{H}{N}\right)}^{2}$ of size *N* × *N*. The cosine basis function $W_{\xi_{1},\xi_{2}}$ of size *N* × *N* at frequency $\xi_{1},\xi_{2}$ is as follows:

(1) $$W_{\xi_{1},\xi_{2}}\left(i,j\right)=c\left(i\right)c\left(j\right)cos[\frac{\left(i+0.5\right)\pi \xi_{1}}{N}]cos\left[\frac{\left(j+0.5\right)\pi \xi_{2}}{N}\right],\;i,j,\xi_{1},\xi_{2}=0,1,\ldots ,N-1\;c(k)=\{\begin{array}{ll} \sqrt{1/N}, & \;\;k=0\\ \sqrt{2/N\;}, & \;\;k=1,2,\ldots ,\;N-1 \end{array}$$

The DCT transform $F_{b}\left(\xi_{1},\xi_{2}\right)$ on block *f_b_*(*x*_1_,*x*_2_) is also a *N* × *N* matrix which is calculated by

(2) $$F_{b}\left(\xi_{1},\xi_{2}\right)=\overset{N-1}{\underset{x_{1}=0}{\sum }}\overset{N-1}{\underset{x_{2}=0}{\sum }}f_{b}\left(x_{1},x_{2}\right)\times W_{\xi_{1},\xi_{2}}\left(x_{1},x_{2}\right)$$

Due to the orthogonality and symmetry of the DCT basis, the inverse DCT (IDCT) transform is given by

(3) $$f_{b}\left(x_{1},x_{2}\right)=\overset{N-1}{\underset{\xi_{1}=0}{\sum }}\overset{N-1}{\underset{\xi_{2}=0}{\sum }}F_{b}\left(\xi_{1},\xi_{2}\right)\times W_{\xi_{1},\xi_{2}}\left(x_{1},x_{2}\right)$$

In this study, we set *N* = 8. In order to pack the DCT operation and IDCT operation into the proposed deep learning model, the DCT transform was wrapped into a 2D convolution operation with 64 filters $\left\{W_{0,0},W_{0,1},\ldots ,W_{7,7}\right\}$ of size 8 × 8. This enabled us to reasonably arrange the high-frequency component and low-frequency component. We conducted a zig-zag pattern in JPEG [31] to reorder the filters as shown in the top–left of Figure 2. In addition, the inverse DCT transform was converted into a 2D transposed convolution with the same filters. Both the convolution operation in the DCT layer and IDCT layer have a stride of 8 to prevent the image patches overlapping. The parameters of these filters are trainable in the training phase.

**Figure 3:**
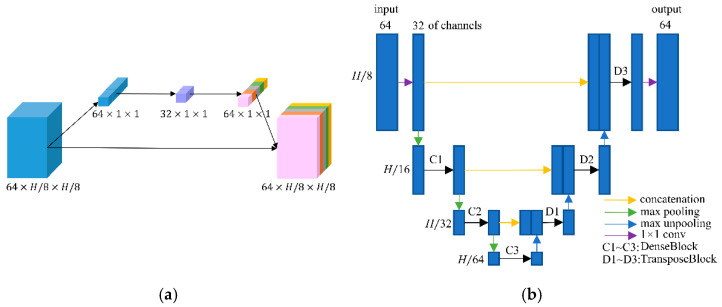
(**a**) Network structure of the frequency-attention block; and (**b**) network structure of the reconstruction block.

After transforming the input image into the DCT domain, frequency features of size 64 × *H*/8 × *H*/8 were obtained. As we knew, the low-frequency component is a comprehensive measure of the intensity of the whole image while the high-frequency component contains the information of the edge and contour. Therefore, low-frequency feature restoration is essential to intensity recovery while high-frequency feature reconstruction plays an important role in edge enhancement. In addition, different degraded images have distinctive characters on each frequency, i.e., low degraded images have more reliability on high frequencies than high degraded images. To separately operate the frequency components according to the degradation levels, a frequency-attention block was applied to explicitly instruct the network to pay attention to the degradation variation. Inspired by the SE block [32], the 64 frequency features were first squeezed into a 64-dimensional vector through global average pooling on each frequency. Then, a fully connected layer followed by a rectified linear unit (ReLU) was utilized to compress the feature space to 32 dimensions. After that, the compressed feature was extended to 64 dimensions as a frequency weight vector by a fully connected layer with a sigmoid operation. The sigmoid function constrains the feature value into 0~1 which represents the proportion of the corresponding frequency component. Finally, the frequency weight vector was expanded to the size of the input frequency feature and multiplied to the input frequency feature as the output of the frequency-attention block. The structure of the frequency-attention block is shown in Figure 3a.

The reconstruction block takes the weighted frequency feature as input and aims to restore the ideal frequency components. Recently, DD-Net [24] has made extraordinary achievements in sparse-view CT reconstruction, contributing to its DenseNet and Deconvolution modules. Inspired by DD-Net, we used the same network structure as DD-Net but made several modifications in our reconstruction block. Firstly, due to the fact that the input of the reconstruction block is not a degraded image of size *H* × *H* but a frequency feature of size *H*/8 × *H*/8 with 64 channels, the receptive field is enlarged and using a 4-fold down-sampling operation seems inappropriate. Therefore, our reconstruction block contains 3 times max pooling and 3 times max un-pooling. Secondly, to achieve the balance between the effectiveness and the number of training parameters, the convolution layer with kernel 5 was replaced by the convolution operation with kernel 3, and the output of each layer had 32 feature maps instead of 16 in DD-Net. These parameters were experimentally determined as seen in Section 3.4. The structure of the reconstruction block is shown in Figure 3b. Since the output of the reconstruction block is an estimate of ideal frequency components, the IDCT layer finally inverses the frequency feature into the image domain. The output of the frequency-domain module is a preliminary prediction of the ideal CT image.

### 2.3. Image-Domain Module

To further improve the textural details and edge information of CT images, an image-domain module was developed to enhance the frequency-domain result by a spatial-attention block and a refining block.

**Figure 4:**
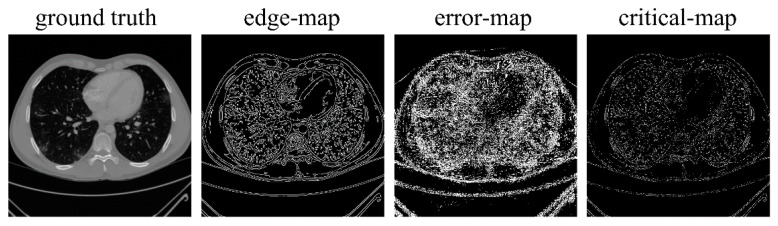
Examples of the ground-truth image, edge-map, error-map and ideal critical-map.

**Figure 5:**
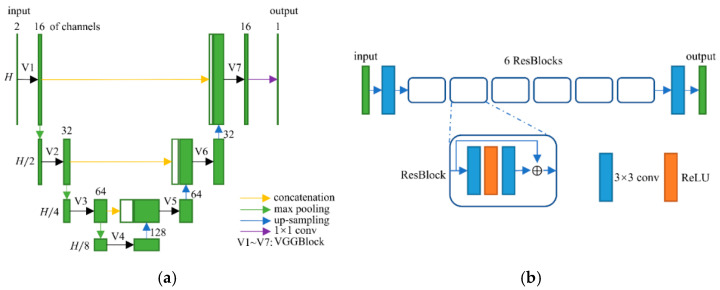
(**a**) Network structure of the spatial-attention block; and (**b**) network structure of the refining block.

Since the reconstruction difficulty of CT images and the visual performance of CT images in the image domain vary with the degree of degradation, the spatial attention block takes the corrupted CT images and the frequency-domain results as input and predicts a critical-map that highlights the edge pixels with large reconstruction errors. Inspired by the critical pixel mask [33], the ideal critical-map is the intersection of an edge-map and an error-map. The edge-map was detected by a Canny operation from the ground-truth CT image. For the error-map, we first calculated the res-map which is the absolute value of the difference between the frequency-domain result and the ground-truth CT image (both normalized to 0~1). Then, we set the pixel value greater than 0.01 to 1 and the rest of the positions to 0. An example of the edge-map, error-map and ideal critical-map is shown in Figure 4. In the inference process, each value of the critical-map represents the probability that the corresponding pixel is a critical pixel. The structure of the spatial-attention block is also a U-Net [26], as shown in Figure 5a. A VGGBlock with two convolutional layers was used to learn the feature in the same feature size level. Finally, the frequency-domain result and the critical-map were concatenated as the input of the refining block, which was composed of 6 ResBlocks to recover the structure details of CT images. Down-sampling operations were not used in this block and the output of each layer has 64 feature maps. The details of the spatial-attention block and refining block are shown in Table S1 (Supplementary Materials) and Figure 5b. In addition, several experiments of parameter selecting were performed which are described in Section 3.4.

### 2.4. Datasets

The training, validation and test datasets consist of 9203 images (1580 brain, 2266 abdomen, 1251 esophagus and 4106 lung), 300 images (53 brain, 74 abdomen, 41 esophagus and 132 lung) and 1000 images (173 brain, 247 abdomen, 136 esophagus and 444 lung), respectively, with different images, all collected from The Cancer Imaging Archive (TCIA) [29,30,34,35,36]. To simulate the real degradation process of the projection data, the original DICOM CT data *hu* (Hounsfield unit (*HU*)) was first converted into an attenuation coefficient μ (${\mathrm{mm}}^{-1}$):

(4) $$\mu =\mu_{water}\times \left(\frac{hu}{1000}+1\right)$$

where $\mu_{water}$ is the attenuation coefficient of water which is approximately 0.02 ${\mathrm{mm}}^{-1}$ at 60 keV. According to the Lambert–Beer law, the received noise-free photons *I* on the detector along ray *l* is given by

(5) $$\;I_{l}=I_{0}\times e^{-p_{l}}$$

where $I_{0}$ is the mean number of photons from the source which is set to $1.0\times 10^{5}$ [37,38,39] and $p_{l}$ represents the linear integral of the attenuation coefficients μ along ray *l*. The real photons ray $\tilde{I_{l}}$ is degraded by the Poisson photon noise and Gaussian electronic noise [15]:

(6) $$\tilde{I_{l}}=Poisson\left\{I_{l}\right\}+n$$

where n represents the additive Gaussian noise with zero mean and a variance of 10. Therefore, the noised integral of the attenuation coefficient is calculated by

(7) $$\tilde{p_{l}}=-\log\left(\tilde{I_{l}}/I_{0}\right)$$

In this study, the Operator Discretization Library (ODL) [40] was used to construct fan beam projection geometry and produce sinogram data $\tilde{p}$. The distance between the source and rotation center was 346 mm, and the distance between the rotation center and detector was 261 mm. The diameter of the field-of-view was 370 mm and the resolution of the CT image was 0.5 mm per pixel. There are 1024 bins in the detector with a resolution of 0.75 mm per pixel. In the training process, the ground truth CT data are randomly projected with 60, 120, 240 views and the FBP algorithm was employed to generate the degraded image of size 512×512.

### 2.5. Network Training

We designed a step-by-step training strategy to gradually learn the mapping from the degraded image to the ground truth image. Firstly, the frequency-domain model was trained to obtain a rough prediction of the ideal image using the mean square error (MSE) loss. Secondly, we independently trained the spatial-attention block using the ideal critical-map as the label image, which was generated from the ground truth image and the reconstruction result of the frequency-domain model. Binary cross entropy (BCE) loss was adopted to classify the critical pixels. Thirdly, the refining block was trained to finely estimate the ground-truth image using mean absolute error (L1) loss. Compared to MSE loss, L1 loss minimizes the absolute differences between the prediction and the ground truth which has the advantage of recovering structure details and enhancing the edge contour [41,42]. Finally, we froze the parameters of the spatial-attention block and conducted the overall training of the frequency-domain model and the refining block using L1 loss.

All modules were trained on a server with one GeForce GTX 1080 Ti using the PyTorch [43] deep learning framework. The batch size was set to 32, 4, 8 and 1, respectively, for the training of the frequency-domain model, spatial-attention block, refining block, and the overall training. The ADAM [44] optimizer was adopted to perform gradient updates with $\beta_{1}=0.9$, $\beta_{2}=0.999$. The initial learning rate was $1.0\times 10^{-4}$ and decreased by half for every $1.0\times 10^{5}$ iteration of training. The pretrained model is available at https://github.com/sunchang2017/degradation-aware-sparse-CT-reconstruction (accessed on 20 October 2021).

## 3. Results

### 3.1. Degradation-Aware Ability Exploration

**Figure 6:**
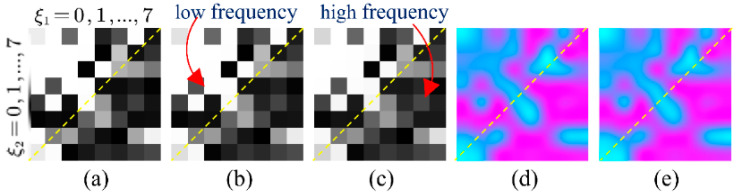
(**a**) Average frequency-attention-map on 60 views; (**b**) average frequency-attention-map on 120 views; (**c**) average frequency-attention-map on 240 views; (**d**) subtraction of average frequency-attention-map on 120 views and 60 views; and (**e**) subtraction of average frequency-attention-map on 120 views and 60 views. In (**d**,**e**), values greater than 0 are shown in pink and values less than 0 are shown in blue.

To analyze the explicit attentional transformations of the frequency-domain module for different degradation levels, we restored the output of the frequency-attention block to the pre-zig-zag order and reshaped it to 8 × 8 as a frequency-attention-map (FAP). The mean of FAP on test datasets of 60 views, 120 views and 240 views are shown in Figure 6a–c. The top of the orange dashed line is the low-frequency part and the bottom is the high-frequency part. We can see that since the low frequencies contain the main information of the CT images, the attention weight of the low frequencies is generally greater than that of the high frequencies. To further explore the specificity of FAP for different degradation levels, the subtraction of FAP on 120 views and 60 views, and the subtraction of FAP on 240 views and 120 views are shown in Figure 6d,e, respectively. It can be intuitively seen that as the degradation level decreases, the weights of high frequencies in the FAP show an overall increasing trend (pink color), while the weights of low frequencies show an overall decreasing trend (blue color), indicating that the network senses an increase in the reliability of the data at a high frequency.

**Figure 7:**
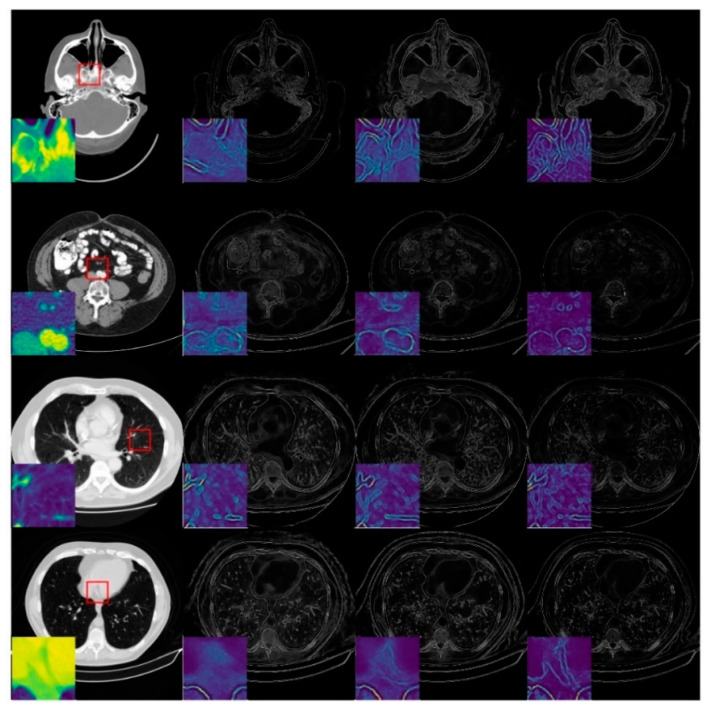
Examples of predicted critical-maps at 60 views, 120 views and 240 views.

To investigate the degradation perception of the image-domain module, we compared the predicted critical-map of the spatial-attention block for the same image with different degradation levels, as shown in Figure 7. The first column is the ideal CT image, whilst the second, third and fourth columns represent the predicted critical-maps at 60 views, 120 views and 240 views, respectively. We zoomed in on the red square area and displayed it in color to observe the textual details. The edge information of the critical-map increases as the degradation decreases, which indicates that the reconstructed values of the contours are generally inaccurate and the later refining block should enhance the detail recovery and reconstruction. In the case of the large degradation level, the critical-map of the image is blurry, indicating that there are still small artifacts interfering in the flat region, which should be further removed in the refining block to improve the overall intensity recovery.

### 3.2. Reconstruction Performance

We compared our method with two non-deep learning methods: FBP and SART [10]; and four deep learning methods: Improved GoogLeNet [28], Tight frame U-Net [27], RED-CNN [25] and DD-Net [24]. For a fair comparison, all four models were re-trained using the same dataset as our method. In addition, we also trained each model with three sets of parameters using training datasets of 60 views, 120 views and 240 views, respectively. We denoted these methods by Improved GoogLeNet+, Tight frame U-Net+, RED-CNN+ and DD-Net+. The number of parameters of these models was three times higher than the original model, as shown in Table 1, where FDM denotes the proposed frequency-domain module. Table 1 also displays the average computational cost (in GPU) of these methods on 1000 images with a size of 512×512. It can be seen that RED-CNN achieves the lowest computational cost. Due to the designed frequency-domain and spatial-domain module, our method has the largest computational cost. However, the reconstruction speed of our method is still comparable with DD-Net.

PSNR and SSIM are used to quantitatively measure the reconstruction algorithms which are defined as

(8) $$\mathrm{MSE}=\frac{1}{mn}\overset{m-1}{\underset{i=0}{\sum }}\overset{n-1}{\underset{j=0}{\sum }}{\left[I\left(i,j\right)-K\left(i,j\right)\right]}^{2}$$

(9) $$\mathrm{PSNR}=10\log\left(\frac{MAX^{2}}{MSE}\right)$$

(10) $$\mathrm{SSIM}=\frac{\left(2\mu_{x}\mu_{y}+c_{1}\right)\left(2\sigma_{xy}+c_{2}\right)}{\left(\mu_{x}^{2}+\mu_{y}^{2}+c_{1}\right)\left(\sigma_{x}^{2}+\sigma_{y}^{2}+c_{2}\right)}$$

where I(i,j) and K(i,j) represents the predicted CT image and the ideal CT image with size m×n. MAX is the maximum pixel value of the image. $\mu_{x},\;\mu_{y}$ is the mean value of I(i,j) and K(i,j). $\sigma_{x},\;\sigma_{y},\;\sigma_{xy}$ is the variance of I(i,j), K(i,j) and the covariance of I(i,j) and K(i,j) respectively. $c_{1}={\left(k_{1}L\right)}^{2}$, $c_{2}={\left(k_{2}L\right)}^{2}$ where L is the range of pixel values, $k_{1}=0.01,\;k_{2}=0.03$.

**Figure 8:**
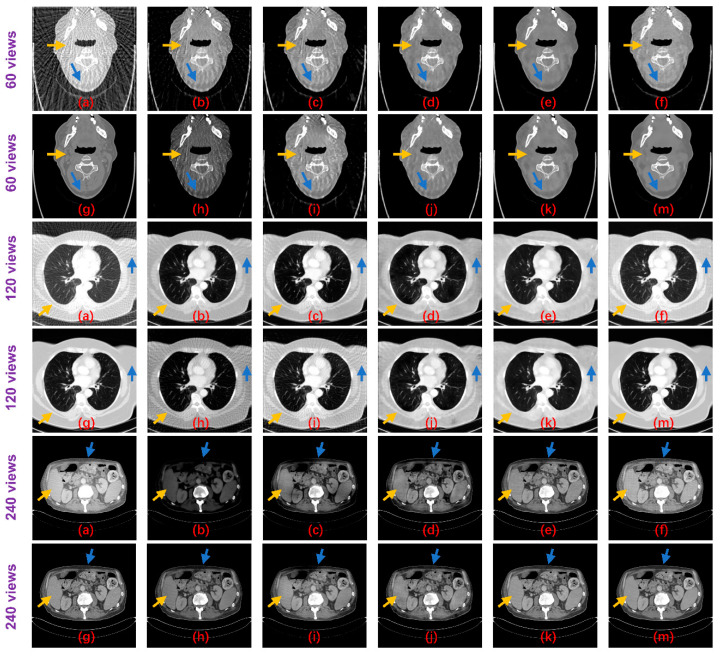
Result images of the proposed method and the compared algorithms (blue and yellow arrows point out the detailed structural differences); (**a**) FBP; (**b**) Improved GoogLeNet; (**c**) Tight frame U-Net; (**d**) RED-CNN; (**e**) DD-Net; (**f**) frequency-domain module (FDM); (**g**) ground truth; (**h**) improved GoogLeNet+; (**i**) tight frame U-Net+; (**j**) RED-CNN+; (**k**) DD-Net+; and (**m**) ours.

Table 2 shows the PSNR and SSIM results of these methods on the test datasets. Both non-deep learning methods performed worse than the deep learning methods, while for the deep learning algorithms, the Tight frame U-Net achieves higher PSNR and SSIM performance compared to Improved GoogLeNet with 25 times the number of parameters. The RED-CNN achieves better PSNR performance than the Tight frame U-Net, especially on the 60 views dataset and the network parameters are only 6% of the Tight frame U-Net, which we believe may be overfitted. Thanks to DenseNet and deconvolution’s ability to capture deep features of images, DD-NET obtains a better PSNR performance than RED-CNN, especially on the head and abdomen datasets, and higher SSIM, especially on the head and esophagus datasets. Particularly, our method outperforms all methods by achieving optimal PSNR and SSIM performance on all datasets. From the perspective of the degradation level, the PSNR of our method is on average 0.84 dB, 0.89 dB and 1.22 dB higher than DD-Net, respectively, for the 60 views, 120 views and 240 views datasets, and the SSIM results are on average 0.02, 0.01 and 0.01 higher, respectively. In terms of the body part, the PSNRs of our method are 0.89 dB, 0.94 dB, 1.29 dB and 0.80 dB higher than those of DD-Net for the head, abdomen, lung and esophagus, respectively, and the SSIM results are 0.02, 0.01, 0.02 and 0.01 higher, respectively. Figure 8 shows the reconstruction results of these methods. It can be seen that the artifact removal performance and detailed retention performance of our proposed method are optimal for all kinds of sparse-view datasets.

**Figure 9:**
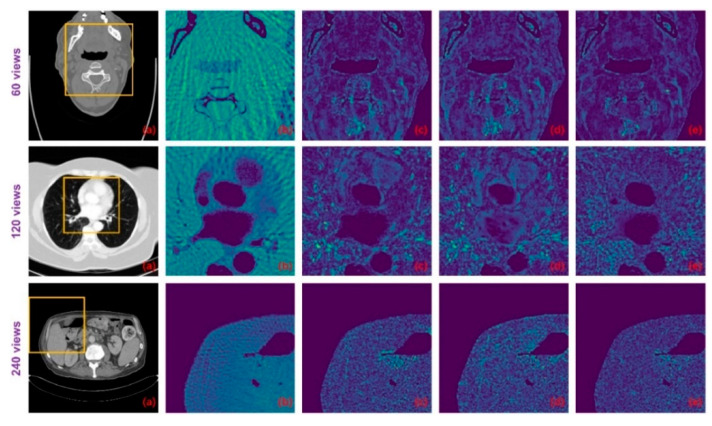
The difference images between the ground-truth CT images and the images resulting from different methods: (**a**) ground truth; (**b**) FBP; (**c**) DD-Net; (**d**) DD-Net+; and (**e**) ours.

For the networks that contain three sets of parameters corresponding to different degradation levels, Improved GoogLeNet+ has a PSNR advantage over Improved GoogLeNet on 60 views and 120 views while SSIM has an advantage only on 60 views. The performance of Tight Frame U-NET+ is generally weaker than that of Tight Frame U-NET, which may be due to the fact that the training dataset of Tight Frame U-NET+ only targets one degradation level, while the training set of Tight Frame U-NET contains three types of degradation, which moderates the overfitting problem to some extent. The average performance of Red-CNN+ and DD-NET+ is better than that of Red-CNN and DD-NET, but still worse than that of our model and more parameters are used. The standard deviations of PSNR and SSIM for our method on the test dataset are shown in Table 3. Suppose the PSNR and SSIM results are both independent samples from a normally distributed population, Table 3 also displays the 95% confidence intervals for PSNR and SSIM results on the test dataset. Figure 9 displays the difference images between the result images and the ideal CT images. It can be seen that the proposed method can reduce the overall intensity error compared to other methods, therefore, achieves better visual performance.

To further evaluate the effectiveness of the proposed method compared to other’s deep learning methods, statistical significance testing is conducted on each method. In particular, we compared the PSNR and SSIM results between the proposed method and other deep learning-based methods to see whether there was a significant difference. The process of significance testing is as follows:

Suppose that *n* pairs of results $\left\{(X_{1},Y_{1}),\ldots ,(X_{i},Y_{i}),\ldots ,(X_{n},Y_{n})\right\}$ are independent, where $X_{i}$ is the PSNR result of the proposed method on the *i*th test image and $Y_{i}$ is the PSNR result of the compared method on the *i*th test image. Then, $\left\{D_{1},\ldots ,D_{i},\ldots D_{n}\right\}$ are independent and can be considered to be from the same distribution, where $D_{i}=X_{i}-Y_{i}$. Assuming that $D_{i}\sim \left(\mu_{D},\;\sigma_{D}^{2}\right),\;i=1,\ldots ,n$ follows a normal distribution, the two-sided null hypothesis $H_{0}$ is that there is no difference in the PSNR result between the proposed method and the compared method. Therefore, the null hypothesis $H_{0}$ and the alternative hypothesis $H_{a}$ can be formulated as follows:

(11) $$H_{0}:\;\mu_{D}=0\;,H_{a}:\mu_{D}\neq 0$$

Then, the significance test is known as the t-test [45] and the test statistic *t* is computed as follows:

(12) $$t=\frac{\bar{d}}{s_{D}/\sqrt{n}}$$

where $\bar{d}$ and $s_{D}$ are the mean and the standard deviation of $\left\{D_{1},\ldots ,D_{i},\ldots D_{n}\right\}$. The *p*-Value *p* is calculated by

(13) p=2×tcdf(−|t|)

where tcdf(·) represents the cumulative distribution function of the t-distribution [45]. We can use *t* and *p* to evaluate the difference between the proposed method and the compared method. The null hypothesis $H_{0}$ is rejected if the t-score is in the critical region or the *p*-Value is less than a predetermined level. The procedure for the significance testing of the SSIM result is the same as the above. Only at this time is $X_{i}$ is the SSIM result of the proposed method on the *i*th test image and $Y_{i}$ is the SSIM result of the compared method on the *i*th test image.

**Figure 10:**
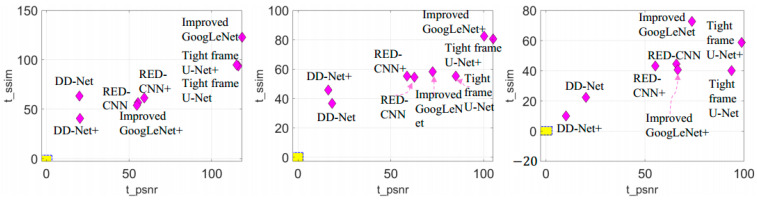
T-score of different deep learning-based reconstruction methods. *t_psnr* and *t_ssim* represent the t-score of the PSNR result and SSIM result, respectively. From left to right are the results of the test datasets of 60 views, 120 views and 240 views. Outside of the yellow area is the critical region (significance level α=0.005).

We performed statistical significance testing on different deep learning-based reconstruction methods on test datasets with 1000 images. Given a significance level α=0.005, the *p*-Values of these methods are all smaller than $1.0\times 10^{-16}$, indicating that we reject $H_{0}$ in favor of $H_{a}$. Table 4 shows the t-score results of these methods. *t_psnr* and *t_ssim* represent the t-score of the PSNR result and SSIM result, respectively. It can be seen that DD-Net and DD-Net+ have relatively small t-scores compared to other methods, while Improved GoogLeNet, Improved GoogLeNet+, Tight frame U-Net and Tight frame U-Net+ have relatively large t-scores on all test datasets. In order to further analyze the differences between these methods, Figure 10 shows the t-scores of these methods on the test datasets. Given a significance level α=0.005, the critical region is |t|≥2.8133 (outside of the yellow region in the figure). It can be seen that the t-scores of all the compared methods are in the critical region of all 60 views, 120 views and 240 views’ test datasets, therefore, the null hypothesis $H_{0}$ is rejected at the chosen level of significance α. Tight frame U-Net+ and Tight frame U-Net+ has the worst performance results. Improved GoogLeNet+ has a fluctuating performance on the test datasets of 60 views, 120 views and 240 views. On the other hand, RED-CNN, RED-CNN+, DD-Net, DD-Net+ have relatively stable results on all datasets but are still in the critical region.

### 3.3. Ablation Study

**Figure 11:**
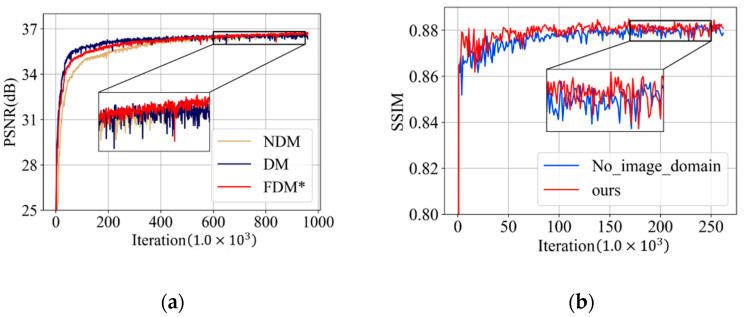
(**a**) PSNR (dB) results on the validation dataset of the ablation experiment on the frequency-domain module; and (**b**) SSIM results on the validation dataset of the ablation experiment on spatial-attention block.

To investigate the effect of the proposed frequency-domain module, we designed two model variants—*NDM* and *DM*. Compared to our frequency-domain module, *DM* discards the frequency-attention block and *NDM* further does not use the DCT weights to initialize the DCT layer and IDCT layer. To make a fair comparison, *NDM* and *DM* were trained using the same strategies as our frequency domain module. The average PSNR results on the validation dataset during the training process are plotted in Figure 11a, where FDM* denotes the proposed frequency-domain module without the final overall training. Table 5 also illustrates the quantitative results. It can be seen that FDM* outperforms *DM* in terms of PSNR, indicating that the frequency-attention block is beneficial in tackling CT images with multiple degradation levels and achieve desirable performance in intensity recovery. FDM* also has an advantage in PSNR compared to *NDM* which demonstrates that learning in the frequency domain is more suitable for understanding the characteristics’ information of the degradation level and yield appealing reconstruction results.

To explore the effect of the spatial-attention block, we design a comparison model *No_image_domain*, which only retains the frequency-domain module and the refining block. In the training phase, we pre-train these two modules, respectively, and then conduct overall training of all parameters. The average SSIM values of all the CT images in the validation dataset are plotted in Figure 11b. Table 6 also illustrates the quantitative results. Our method achieves better results in terms of SSIM on the validation datasets which illustrates that the spatial-attention block has the advantage of preserving structural details and textural features.

### 3.4. Network Parameter Tuning

**Figure 12:**
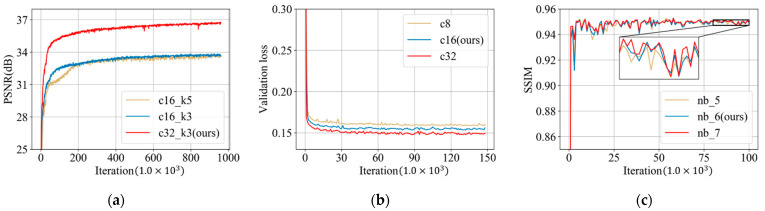
(**a**) PSNR (dB) results from the validation dataset during the training of the reconstruction block (*c16_k5* represents a reconstruction block with 16 feature maps and convolution kernels of size 5); (**b**) loss on the validation dataset during the training of the spatial-attention block (*c8* represents 8 channels in the spatial-attention block); and (**c**) SSIM results on the validation dataset during the training of refining block (*nb_5* represents the refining block with 5 ResBlocks).

There are several parameters that need to be optimized in the reconstruction block, spatial-attention block and refining block. Figure 12a displays the average PSNR results of images in the validation dataset during the training of the reconstruction block. In this figure, *c16_k5* represents a reconstruction block with 16 feature maps and convolution kernels of size 5. It can be clearly seen that the PSNR increases considerably from *c16* to *c32*, while a large kernel size *k5* has little improvements on PSNR. Therefore, we construct the reconstruction block with parameter *c32_k3*.

For the number of channels in the spatial-attention block, we change this parameter into three values: 8, 16 and 32. The validation loss during the training of the spatial-attention block is shown in Figure 12b. It can be seen that as the number of channels increases from 8 to 32, the performance becomes better. Considering the trade-off of the model size and the reconstruction performance, value 16 was selected as the number of channels in the proposed spatial-attention block.

As for the refining block, we conducted three variant models with a different number of ResBlocks: 5, 6 and 7. Figure 12c displays the SSIM results of these models during the training phase. It can be seen that all models have a similar performance after convergence, however, and the network with seven ResBlocks has the overall largest SSIM result, followed by the model with six ResBlocks and the SSIM result of the network with five ResBlocks is relatively lower than the others. Therefore, considering the trade-off of the model size and the reconstruction performance, we determined this parameter as six in our proposed network.

## 4. Discussion

In this study, we developed a single deep learning-based framework to improve the performance of sparse-view CT reconstruction on multiple degradation levels. Previous deep learning-based methods fail to achieve satisfactory results on different degradation levels due to the training on a single degradation level. Inspired by the distinctive frequency features of different degradation levels, as shown in Figure 1, the proposed framework was trained on datasets with different degradation levels, particularly, a frequency-domain module and an image-domain module were devised to improve the effectiveness of the deep learning network.

Experimental results shown in Figure 6 indicate that the proposed frequency-attention block was able to capture the characteristics of a different degradation level in the frequency domain. This result coincides with the previous conclusion of Figure 1, which demonstrates that the frequency-attention block is able to differentiate degradation levels and adaptively adjust the frequency-attention-map to better guide the reconstruction block. In addition, as shown in Figure 7, the spatial-attention block can sense the specificity of different degradation levels in the image domain, and give the pixels that should be focused on the later reconstruction to further map the ground truth image.

As for the reconstruction performance results in Table 2, both non-deep learning methods FBP and SIRT performed worse than deep learning methods, indicating that supervised learning can better learn the prior distribution of real CT images, which is beneficial for solving the inverse problem. However, the performance of the previous deep learning models degrades due to the gap in the degradation level between the training datasets and test datasets. One reasonable explanation is that these methods do not manually include degradation knowledge as a priori or as degradation-aware modules especially designed to explicitly learn degradation levels. Directly expanding the dataset with all degradation levels may produce a rather compromising result. This method of letting the network learn degradation priori implicitly without improvements in model design produces unstable effects. Therefore, it is promising for future work to design more efficient and robust degradation-aware modules.

With the explicit learning of degradation levels in both the frequency and image domain, our method outperforms all the deep learning-based methods in terms of different degradation levels and body parts, as well as achieves a satisfactory trade-off between the size of the network and the performance (Table 1). Moreover, the statistical significance of the testing results (Table 4 and Figure 10) demonstrates that the differences in the PSNR and SSIM results between the proposed method and other methods are statistically significant. In addition, our method achieves better visual results with more textual structure details and less reconstruction error (Figure 8 and Figure 9).

In addition, the advantage of our model lies in its extensibility. In the context of more degradation levels, the parameters of Red-CNN+ and DD-NET+ will increase exponentially, while the parameters of our model do not need to increase, as only the degradation species of the dataset needs to be extended, which makes our model more advantageous in practice.

## Figures and Tables

**Table 1:** Number of parameters and computational cost of different deep learning-based methods.

Method	Number of Parameters	Computational Cost (per Image)
Improved GoogLeNet	1.25 M	0.0032 s
Tight frame U-Net	31.42 M	0.0034 s
RED-CNN	1.85 M	0.0010 s
DD-Net	0.56 M	0.0057 s
FDM	0.92 M	0.0050 s
Improved GoogLeNet+	3.75 M	0.0032 s
Tight frame U-Net+	94.26 M	0.0034 s
RED-CNN+	5.55 M	0.0010 s
DD-Net+	1.68 M	0.0057 s
ours	1.63 M	0.0062 s

**Table 2:** Quantitative results of PSNR (dB)/SSIM on different reconstruction algorithms. The best results on each dataset are marked in bold.

Views	Body Part	FBP	Improved GoogLeNet	Tight Frame U-Net	RED-CNN	DD-Net	FDM
60	Head	15.9478/0.3516	21.6936/0.4607	25.0216/0.6051	31.0674/0.8171	35.4189/0.9147	33.8139/0.8088
	Abdomen	14.6239/0.3977	19.5052/0.4886	25.3207/0.6907	32.2187/0.8760	35.9957/0.9237	35.8076/0.9028
	Lung	15.7114/0.4177	21.1298/0.5315	25.1913/0.6981	30.2601/0.8481	33.9357/0.9080	33.4616/0.8532
	Esophagus	13.8681/0.3428	18.2902/0.4173	24.0947/0.6291	31.9415/0.8446	35.6552/0.9220	34.8013/0.8327
120	Head	20.5276/0.4536	32.3011/0.7400	34.5627/0.8666	34.5000/0.8859	39.4975/0.9488	36.5722/0.8386
	Abdomen	18.6596/0.4940	29.1715/0.7059	33.0186/0.9035	35.2896/0.9210	39.3417/0.9496	38.4492/0.9184
	Lung	20.0424/0.5333	29.9168/0.7671	31.6234/0.8894	33.0130/0.8990	37.0283/0.9388	36.7272/0.8877
	Esophagus	17.2796/0.4285	27.0452/0.6057	32.2867/0.8630	34.7532/0.8904	38.8522/0.9518	37.1417/0.8544
240	Head	26.5085/0.5865	32.4706/0.8255	36.7117/0.9150	37.1478/0.9052	42.6465/0.9660	38.4318/0.8443
	Abdomen	25.4890/0.6368	31.2142/0.8480	34.9420/0.9465	36.7007/0.9384	41.8674/0.9654	41.0545/0.9425
	Lung	25.6982/0.6791	29.5797/0.8113	33.2487/0.9263	35.1415/0.9260	39.1089/0.9560	38.6735/0.9058
	Esophagus	23.0284/0.5516	34.1418/0.8526	34.6916/0.9156	37.3571/0.9097	41.3469/0.9687	39.7422/0.8852
**Views**	**Body Part**	**SART**	**Improved GoogLeNet+**	**Tight Frame U-Net+**	**RED-CNN+**	**DD-Net+**	**Ours**
60	Head	23.4269/0.6964	28.0190/0.7486	25.8151/0.6176	31.4201/0.8158	35.5668/0.9256	**36.0998/0.9421**
	Abdomen	18.4496/0.6381	28.3975/0.7401	25.3411/0.6758	32.1736/0.8687	36.0104/0.9338	**36.8327/0.9434**
	Lung	17.1589/0.6097	27.2493/0.7354	25.6127/0.6899	30.1739/0.8490	33.7070/0.9152	**34.9458/0.9291**
	Esophagus	18.5389/0.6526	29.2958/0.7119	24.2923/0.6177	31.7161/0.8389	35.1918/0.9291	**36.4700/0.9428**
120	Head	28.6282/0.7748	30.8313/0.7143	30.0908/0.6645	35.4605/0.8824	39.5932/0.9517	**40.2681/0.9630**
	Abdomen	23.1838/0.7338	28.2309/0.6705	27.7061/0.6966	35.7323/0.9180	39.4556/0.9521	**40.1679/0.9606**
	Lung	21.6494/0.7158	27.6462/0.6789	28.7425/0.7372	33.3552/0.9009	37.1235/0.9433	**38.3021/0.9525**
	Esophagus	22.7435/0.7335	26.2278/0.5736	25.9611/0.6196	35.0883/0.8977	39.0283/0.9550	**39.5306/0.9625**
240	Head	34.5998/0.8424	35.8046/0.8199	34.5272/0.7890	38.4972/0.9242	43.3962/0.9727	**43.8769/0.9755**
	Abdomen	29.7504/0.8250	35.0612/0.8505	32.7387/0.8281	37.7709/0.9488	42.3253/0.9712	**43.0279/0.9724**
	Lung	27.7240/0.8141	33.6383/0.8704	31.9236/0.8353	35.7299/0.9385	39.9550/0.9641	**40.7020/0.9670**
	Esophagus	28.4788/0.8112	32.4359/0.7369	30.5312/0.7478	37.2861/0.9355	41.8500/0.9743	**42.2515/0.9745**

**Table 3:** Variability measures of the proposed method on the test dataset (at 95% confidence level).

Metric	Views	Standard Deviation	Confidence Interval
PSNR	60	3.0346	(35.8188 ± 0.1883)
	120	3.2308	(39.2702 ± 0.2005)
	240	3.5828	(42.0365 ± 0.2223)
SSIM	60	0.0214	(0.9368 ± 0.0013)
	120	0.0181	(0.9577 ± 0.0011)
	240	0.0164	(0.9708 ± 0.0010)

**Table 4:** T-score of different deep learning-based reconstruction methods. *t_psnr* and *t_ssim* represent the t-score of the PSNR result and SSIM result, respectively.

Compared Method	60 Views		120 Views		240 Views	
	t_psnr	t_ssim	t_psnr	t_ssim	t_psnr	t_ssim
Improved GoogLeNet	118.2965	122.8433	72.5626	58.2919	73.6602	72.7600
Tight frame U-Net	116.1232	93.8206	85.0838	55.3261	93.7110	40.0548
RED-CNN	55.4372	56.7843	62.7069	54.5588	65.8841	44.3951
DD-Net	19.9694	63.3336	18.4503	36.6697	20.1678	22.4695
Improved GoogLeNet+	54.7750	54.1022	100.2554	82.4739	66.5465	40.6918
Tight frame U-Net+	115.1382	94.8125	105.0866	80.6470	98.7682	58.7093
RED-CNN+	59.2286	61.5391	58.8350	55.3430	55.2423	43.2324
DD-Net+	20.3308	40.7363	16.3571	45.8754	10.0287	10.1868

**Table 5:** Quantitative PSNR results of the ablation experiment on the frequency-domain module. FDM* denotes the proposed frequency-domain module without the final overall training. The best results on each dataset are marked in bold.

Views	Body Part	NDM	DM	FDM*
60	Head	32.8563	32.5425	**33.7495**
	Abdomen	32.8446	32.6188	**35.6840**
	Lung	32.9257	32.6978	**33.1673**
	Esophagus	32.8197	32.6037	**33.7173**
120	Head	35.7321	35.5704	**36.5904**
	Abdomen	35.8170	35.6162	**38.1990**
	Lung	35.6267	35.5710	**36.1079**
	Esophagus	35.7173	35.5953	**36.1940**
240	Head	37.6943	37.5759	**38.4270**
	Abdomen	37.9056	37.6344	**40.3256**
	Lung	37.6960	37.4694	**38.2643**
	Esophagus	37.6851	37.3774	**38.1505**

**Table 6:** Quantitative SSIM results of the ablation experiment’s spatial-attention block. The best results on each dataset are marked in bold.

**Views**	**Body Part**	**No_Image_Domain**	**Ours**
60	Head	0.9396	**0.9421**
	Abdomen	0.9415	**0.9434**
	Lung	0.9268	**0.9291**
	Esophagus	0.9404	**0.9428**
120	Head	0.9619	**0.9630**
	Abdomen	0.9596	**0.9606**
	Lung	0.9510	**0.9525**
	Esophagus	0.9612	**0.9625**
240	Head	0.9751	**0.9755**
	Abdomen	0.9719	**0.9724**
	Lung	0.9660	**0.9670**
	Esophagus	0.9739	**0.9745**

## Data Availability

The pretrained model is available at https://github.com/sunchang2017/degradation-aware-sparse-CT-reconstruction (accessed on 20 October 2021).

## Supplementary Materials

The following are available online at https://www.mdpi.com/article/10.3390/tomography7040077/s1, Table S1: The parameters of the spatial-attention block.
