# Supplementary material for: Degradation-Aware Deep Learning Framework for Sparse-View CT Reconstruction
Source: Tomography. 2021 Dec 9;7(4):932–49. doi: 10.3390/tomography7040077 (PMC8704775; doi:10.3390/tomography7040077)
Supplement: Supplementary file 1 [file tomography-07-00077-s001.zip › tomography-1449973-supplementary.pdf]

Table S1. The parameters of the spatial-attention block.

Layers	Parameters	Output Size
V1	conv (k1s1p0)[1]+BN[2]+ReLU	$512 \times 512 \times 16$
	conv (k3s1p1)+BN+ReLU	$512 \times 512 \times 16$
DS	2 × 2 MaxPooling	$256 \times 256 \times 16$
V2	conv (k1s1p0)+BN+ReLU	$256 \times 256 \times 32$
	conv (k3s1p1)+BN+ReLU	$256 \times 256 \times 32$
DS	2 × 2 MaxPooling	$128 \times 128 \times 32$
V3	conv (k1s1p0)+BN+ReLU	$128 \times 128 \times 64$
	conv (k3s1p1)+BN+ReLU	$128 \times 128 \times 64$
DS	2 × 2 MaxPooling	$64 \times 64 \times 64$
V4	conv (k1s1p0)+BN+ReLU	$64 \times 64 \times 128$
	conv (k3s1p1)+BN+ReLU	$64 \times 64 \times 128$
US	UnSampling+concatenation	$128 \times 128 \times (64 + 128)$
V5	conv (k1s1p0)+BN+ReLU	$128 \times 128 \times 64$
	conv (k3s1p1)+BN+ReLU	$128 \times 128 \times 64$
US	UnSampling+concatenation	$256 \times 256 \times (32 + 64)$
V6	conv (k1s1p0)+BN+ReLU	$256 \times 256 \times 32$
	conv (k3s1p1)+BN+ReLU	$256 \times 256 \times 32$
US	UnSampling+concatenation	$512 \times 512 \times (16 + 32)$
V7	conv (k1s1p0)+BN+ReLU	$512 \times 512 \times 16$
	conv (k3s1p1)+BN+ReLU	$512 \times 512 \times 16$
conv	k1s1p0	$512 \times 512 \times 1$

[1] conv(k1s1p0) represents the convolutional layer with kernel size 1, stride 1 and 0 paddings.
[2] batch normalization
